# A whole-of-community approach to promote cardiovascular health: Healthy Communities in Moldova

**DOI:** 10.1093/eurpub/ckac131.324

**Published:** 2022-10-25

**Authors:** F Secula, D Berari, A Curteanu, S Nicolaescu, H Prytherch

**Affiliations:** 1 University of Basel, Basel, Switzerland; 2 Swiss Tropical and Public Health Institute, Basel, Switzerland; 3 Healthy Life project, Swiss Agency for Development and Cooperation, Chisinau, Moldova; 4 Mother and Child Institute, Chisinau, Moldova; 5 Ministry of Health, Chisinau, Moldova; 6 School of Public Health Management, Chisinau, Moldova

## Abstract

A low-income country, Moldova is facing a rapidly growing burden of non-communicable diseases with a cardiovascular disease mortality rate of 202.2 per 100'000. Scarce resources require urgent preventative actions and involvement of communities and patients in the definition and design of locally relevant health promoting activities. Our intervention is a holistic, whole-of-community approach to foster local leadership in health promotion for the prevention of cardiovascular risks. The Healthy Life project to reduce the burden of non communicable diseases, funded by the Swiss Agency for Development and Cooperation (SDC), operates since 2016 in rural communities of Moldova. We present a 5-step model towards initiating Healthy Communities in Moldova based on 5 complementary steps:

1. Raising awareness on cardiovascular risk factors through community education seminars tailored to audience needs

2. Building multisectoral community coalitions for health between local elected leaders and community representatives around local health data and locally defined health priorities

3. Fostering community leadership in defining local health solutions through health asset mapping exercises

4. Community-led small projects based on identified needs and assets to improve local cardiovascular health

5. Empowering chronic patients for healthy behavior change through community-based self-management workshops

The Healthy Communities intervention was rolled out to 40 communities in 20 districts, reaching 1332 direct beneficiaries. Important learning emerged from social accountability mechanisms embedded in community coalitions which ensure regular, health-focused dialogue on community health. Using local assets to health problem solving is changing the perspective on the resourcefulness of community actors in health. Progress towards sustainability is achieved by local public authorities and community making a matching contribution to start-up funding to small projects.

**Key messages:**

• Our Healthy Communities model proposes a strength-based vision of health promotion which empowers community coalitions to self-organize for the prevention of cardiovascular risk factors.

• We promote bottom-up approaches that strengthen local health governance and community leadership into defining and addressing local health priorities.

